# The Potential of Newly Established Grassland Strips and Permanent Semi-Natural Grassland to Promote Common Carabids and Spiders on Arable Land

**DOI:** 10.3390/insects16050439

**Published:** 2025-04-22

**Authors:** Ronnie Walcher, Dominik Rabl, Manuela Bürgler, Raja Imran Hussain, Bea Maas, Bernhard Krautzer, Dietmar Moser, Thomas Frank

**Affiliations:** 1 Department of Ecosystem Management, Climate and Biodiversity, Institute of Zoology, BOKU University, Gregor-Mendel-Straße 33, 1180 Vienna, Austria; ronnie.walcher@boku.ac.at (R.W.); raja.hussain@boku.ac.at (R.I.H.); bea.maas@univie.ac.at (B.M.); 2 Department of Animal Ecology and Tropical Biology, Biocenter, University of Würzburg, Glashüttenstraße 5, 96181 Rauhenebrach, Germany; dominik.rabl@umweltbundesamt.at; 3 Environment Agency Austria, Spittelauer Lände 5, 1090 Vienna, Austria; 4 Department of Botany and Biodiversity Research, University of Vienna, Rennweg 14, 1030 Vienna, Austria; dietmar.moser@univie.ac.at; 5 Institute of Plant Production and Cultural Landscape, Federal Research Institute Gumpenstein, Altirdning 11, 8952 Irdning, Austria; bernhard.krautzer@raumberg-gumpenstein.at

**Keywords:** epigean carabids and spiders, semi-natural grassland, grassland strips

## Abstract

The implementation of grassland strips and maintenance of permanent semi-natural grassland are intended to promote insects on arable land to mitigate the consequences of habitat loss. While effects of grassland restoration on arable land in terms of improving overall insect diversity is well documented, knowledge on the effects on common arable species is deficient. We assessed the abundance of seven epigean carabids and five spiders in newly established grassland strips, adjacent permanent semi-natural grassland and arable crops nearby and further away from grassland strips over six years. Abundance was investigated using pitfall traps. Most carabids showed the highest abundance in arable crops and grassland strips, while permanent semi-natural grassland tended to be avoided. Epigean spiders showed similar patterns, however, some species were also associated with permanent semi-natural grassland. *Pardosa palustris* showed a distribution pattern with the highest individual numbers in OG and decreasing numbers toward the adjacent habitat’s grassland strips and cereals, most likely indicating spillover. The number of *Brachinus crepitans*/*explodens* was significantly higher in the first compared to the last sampling year in grassland strips due to changing habitat conditions with increasing habitat age. In conclusion, grassland strips provide an additional habitat for most of the studied species.

## Simple Summary

Semi-natural grassland and newly established grassland strips potentially support insects in agricultural areas. Carabids and spiders are important predators in agroecosystems and play a crucial role in pest control. We investigated how the most common arable epigean carabids and spiders respond to newly established grassland strips in an Austrian agricultural area and whether these strips provide additional habitat for common arable species. We also investigated whether cereal fields adjacent to grassland strips benefit from higher abundance of arable species. We also analyzed the role of old semi-natural grassland for arable species. While carabids showed a clear preference for cereal fields and grassland strips, old semi-natural grassland habitats were avoided. Although, epigean spiders showed very similar patterns, some were also associated with permanent semi-natural grassland. *Pardosa palustris* used grassland strips to move into adjacent cereal fields. The number of *Brachinus crepitans*/*explodens* was significantly higher in the first sampling year compared to the last sampling year in grassland strips. Further, we obtained colonization of the two spider species *Pardosa palustris* and *Pachygnatha degeeri* between grassland strips and adjacent cereals. We conclude that most of the studied species benefit from grassland strips and that grassland strips can provide additional habitat for common arable species.

## 1. Introduction

The destruction and fragmentation of habitats, intensive agricultural land-use and climate change interact in complex ways and are leading to an unprecedented biodiversity crisis [1]. In particular, the decline of semi-natural habitats has a severe impact on arthropod communities in arable areas, as they represent important habitats such as food sources, shelter and hibernation sites [2,3]. Thus, the maintenance of semi-natural habitats such as road verges, field margins, fallows, permanent semi-natural grassland habitats [4] and the establishment of new flower or grassland strips is important to mitigate the consequences of habitat loss on insect populations [5,6]. Semi-natural grassland, among other semi-natural habitats, play an essential role in maintaining and promoting vital insect populations on arable areas [7] and thus in sustainable agriculture. However, decades of intensive land use and the associated increase in field size have led to a sharp decline in semi-natural grassland habitats in agroecosystems [8].

To mitigate the decline of semi-natural grassland habitats, the implementation of grassland and flower strips has been increasingly applied in the past decades, most commonly as part of agro-environmental schemes [9,10,11,12,13]. A recent study by Hussain et al. [14] provided substantial insights into the effects of newly established grassland strips in an Austrian agricultural area on several insect communities. They connected the grassland strips to permanent long existing semi-natural grassland in the immediate vicinity, to investigate distance effects and thus the potential of this habitat for the promotion of different insect taxa.

Adjacent permanent semi-natural grassland may act as source habitat for a variety of arthropods to invade the grassland strips and crop fields. The present study aims at whether this is also true for common arable epigean carabids and spiders. We considered seven carabid and five spider species which were the most common species in the habitats studied. These highly abundant species were studied because they are potentially important biocontrol agents and were not taken into consideration on a species level so far [14]. We investigated their distribution among newly established grassland strips, permanent semi-natural grassland and cereal fields near and cereal fields far away from newly established grassland strips. We expected newly established grassland strips to be an additional habitat for these common species. Based on the findings of Hussain et al. [14] who considered carabids and spiders on the taxa level, we further expected spill-over effects of common carabid and spider species between newly established grassland strips and cereal fields near, leading to a higher abundance of epigean carabids and spiders in cereal fields near compared to cereal fields far away from grassland strips, and asked whether (i) there is a positive distance effect from old semi-natural grassland into the adjacent newly established grassland strips, cereal fields near and cereal fields far from grassland strips, i.e., resulting in higher numbers and (ii) number of individuals changes across years.

## 2. Materials and Methods

### 2.1. Experimental Setup

We conducted our field work in the eastern part of the Tullnerfeld region (Lower Austria) near the villages of Ollern (48°16′2.6112; 16°4′59.0664) and Elsbach (48°15′4.4316; 16°2′54.3912). The climate is continental, with a mean annual air temperature of 9.6 °C and a mean annual precipitation of about 900 mm [15]. The prevailing climatic and topographical conditions combined with the high soil fertility makes this region one of the most productive regions in Austria. The structural diversity of the study region is heterogenous. A small-scale mixture of conventionally used arable land, permanent semi-natural grassland and forest patches characterize the Tullnerfeld region.

**Figure 1:**
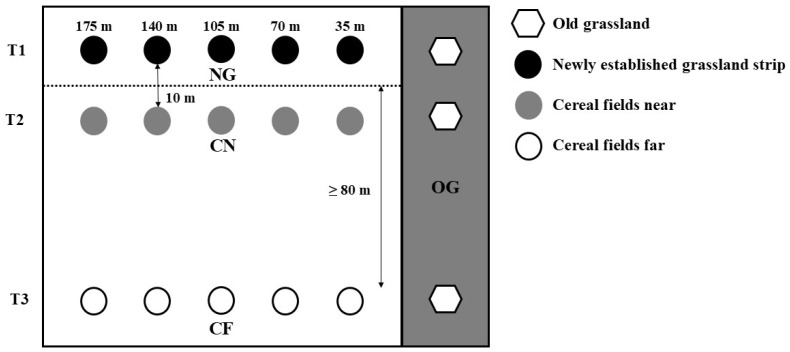
The sketch shows the experimental setup along three transects. OG—old grassland (grey area on the right), NG—newly established grassland strip, CN—cereal field near and CF—cereal field far. White hexagons represent study plots along OG, black circles along NG, gray circles along CN and white circles along CF transect. T1, T2, T3…transects. Sketch modified after Hussain et al. [14].

Our experimental setup comprised five permanent semi-natural grasslands (hereafter designated as OG = old grassland), with transects leading into the adjacent habitat types: five transects into newly established grassland strips (hereafter designated as NG = newly established grassland), five transects into cereal fields near to newly established grassland strips (CN = cereal near) and five transects into cereal fields far away from the newly established grassland strips (CF = cereal far). The cereal fields were planted with winter wheat (*Triticum aestivum* L). Along each transect, we selected six sampling plots with regular distances of 35 m between them, starting from the old grassland (OG) at 0 m. The plots were spaced at intervals of 35 m, extending into the transect up to a distance of 175 m (Figure 1). In total, we studied 15 transects with 6 sampling plots each summing up to 90 sampling plots. The first sampling plot of each transect was located in OG, the further five sampling plots were located in the adjoining transects (CF, CN and NG) (Figure 1, modified after Hussain et al. [14]). The 15 transects were similar in terms of geology, soil characteristics and topography.

The grassland strips were established in August 2016 to guarantee sufficient development of the grassland strips in the consequent sampling years 2017–2022. A mixture of native wild plants was used for seeding, which corresponded to the plant species composition of the OGs. A mixture of 41 native plant species was finally selected for seeding [16]. The grassland strips were sized between 1800 m^2^ and 2500 m^2^. The width of the grassland strips was 10 m. The OGs were extensively managed and mowed twice a year (June and August). The NGs were mowed only once a year (end of July) and the plant material was removed. We set up the traps and finished sampling before mowing. No fertilizers were applied to the OG and NG habitats. All crop fields investigated were managed conventionally and harvested mid-July.

### 2.2. Collection of Carabid Beetles and Spiders

The individual numbers of epigean carabid beetles (*Anchomenus dorsalis* (Linnaeus, 1758), *Bembidion lampros* (Schrank, 1781)/*obtusum* (Fabricius, 1792), *Brachinus crepitans* (Linnaeus, 1761)/*explodens* (Linnaeus, 1758), *Poecilus cupreus* (Linnaeus, 1758), *Pterostichus melanarius* (Illiger, 1798)) and spiders (*Pachygnatha degeeri* (Walckenaer, 1802), *Pardosa agrestis* (Westring, 1861), *Pardosa palustris* (De Geer, 1778), *Oedothorax apicatus* (C.L. Koch, 1839), *Trochosa ruricola* (De Geer, 1778)) were evaluated by using pitfall traps consisting of solid glass vials (5 cm opening diameter, 10 cm depth). Traps were set at ground level with the edge of each trap flush with the ground surface. Each trap was filled up to one-third with a mixture of propylene glycol and water in a ratio of 2:1. An odorless detergent was added to the mixture to break the surface tension of the mixture. A solid metal roof (10 × 10 cm) was placed over each trap in a height of 2–3 cm to protect the traps from being washed out by rain. Sampling was conducted three times between the beginning of April and the beginning of June in each sampling year (2017, 2018, 2019, 2021, 2022; no data available for 2020), at timely intervals of approximately 3 weeks. At each sampling date, traps were open for one week and collected afterwards.

The collected carabid beetles and spiders were sorted, preserved in 70% ethanol and identified to species level using a stereo microscope and identification literature according to Freude et al. [17] and Trautner and Geigenmüller [18]. Spiders were identified to species level according to Nentwig et al. [19]. In total, 19,599 carabid and 19,183 spider individuals were collected. The seven carabid species accounted for 90%, and the five spider species accounted for 70% of the total carabid and spider individuals. *Brachinus* and *Bembidion* species were considered in the complexes *Brachinus crepitans*/*explodens* and *Bembidion lampros*/*obtusum*. Grouping these species into complexes is justified because within the complexes the species have similar habitat requirements and life cycles [20,21].

### 2.3. Statistical Analysis

Analyses were conducted using R Statistical language (version 4.0.4; R Core Team [22]). We examined differences in the number of individuals of epigean carabids and spiders between the habitat types OG, NG, CN and CF, by computing generalized linear mixed models (GLMM) where individual carabid and spider species were analyzed in a separate model. Since we used count data, we specified GLMMs with Poisson error distributions using the lmer-function (R-package lme4; version 1.1.27.1 [23]). Models were checked for overdispersion to ensure accurate modeling of count data with high variability in species counts using the check_overdispersion-function (R-package performance; version 0.10.2, [24]). To account for zero-inflation of the GLMM-models we tested the models with the check_zeroinflation-function of the same R-package. In case of overdispersion we included an observation level random effect as suggested by Harrison et al. [25] or carried out negative binomial GLMM-models. To account for zero-inflation, i.e., when the model was over- or underfitting zeros, we performed generalized linear mixed models using a template model builder applying the function glmmTMB (R-package glmmTMB; version 1.1.3, [26]). Zero-inflation was applied due to a large number of plots with no species captures, ensuring that model results account for sparsely populated habitats.

Habitat type was included as explanatory variable (fixed factor) in the models and the number of individuals of single epigean carabids and spiders was included as dependent variable. Sampling plot nested in transect was included as random factor and additionally sampling time nested in year was added as random factor to account for multiple sampling within years. We used the function glht (R-package multcomp version 1.4.23, [27]) to perform Tukey post hoc tests to detect differences in the abundance of epigean carabids and spiders between the four habitats studied. These pairwise comparisons allowed us to directly test significant differences between habitat types, and the resulting *p*-values are reported in the main text.

Furthermore, we analyzed how individual numbers of epigean carabids and spiders changed among the six sampling years within the habitat types. Therefore, carabid and spider species were included as dependent variables, and sampling year was included as explanatory variable in GLMM-models. Before the analysis, the number of individuals of each species was divided by the total number of individuals in the respective habitat. We checked for overdispersion and zero-inflation and accounted for both with the corresponding models. All graphs were produced using the R-package ggplot2 (version 3.4.4, [28]).

We used generalized linear models (GLM) to test how individual numbers of epigean carabids and spiders changed along the transects from OG to the field interior in CF, CN and NG. Abundance was taken as the response variable, and the four habitat types were specified as fixed variables. The distance along the transects (ranging from 0 m to 175 m) was used to assess the influence of proximity of OG and NG, CF and CN on the abundance of carabid and spider species. This was done to investigate how species distributions varied along the transect from OG to NG, CF and CN. We specified GLMs with Poisson distributions and accounted for overdispersion, which was checked by the function dispersion.test (R-package “AER” 1.2-4, [29]), by specifying quasi-Poisson models.

## 3. Results

### 3.1. Carabids

**Figure 2:**
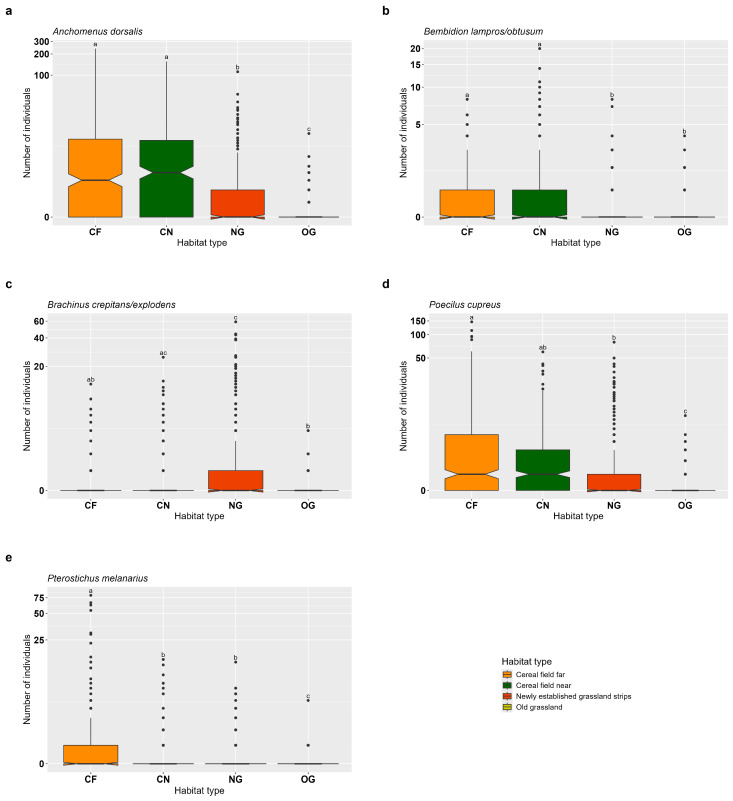
Effects of habitat type on common epigean carabids in old semi-natural grassland (OG), newly established grassland strips (NG), cereal fields near (CN) and cereal fields far (CF). (**a**) *Anchomenus dorsalis*, (**b**) *Bembidion lampros*/*obtusum*, (**c**) *Brachinus crepitans*/*explodens*, (**d**) *Poecilus cupreus*, (**e**) *Pterostichus melanarius*. Box-plots show the median, notches, 25% and 75% percentiles and outliers (●). Indicator letters reveal significant differences between habitat types. Habitat types sharing the same letters are not significantly different (Tukey HSD, significance level *p* < 0.05). The figure is log-transformed on the y-axes. Number of individuals are total count data per sampling plot.

*Anchomenus dorsalis* showed the highest individual numbers in CF and CN, indicating a positive effect of these habitat types on its abundance. Both CF and CN differed significantly from NG and OG (*p* < 0.001), where lower numbers of individuals were recorded. NG and OG were also significantly different from each other, with the lowest abundance observed in OG (*p* < 0.001), suggesting a particularly negative effect of OG on *A. dorsalis* (Figure 2a, Table S1). Similarly, the two *Bembidion* species showed higher abundances in CF and CN, which were both significantly different from NG and OG (*p* < 0.001), where fewer individuals were found. NG and OG did not differ significantly from one another (*p* = 0.817), suggesting a generally lower suitability of these two habitats for the two *Bembidion* species (Figure 2b, Table S1). For *Brachinus* species, the highest number of individuals was found in NG, indicating a positive response in this habitat. However, abundance in NG did not significantly differ from CN (*p* = 0.069), while it was significantly higher than in OG (*p* < 0.001). CF and OG showed similar, lower abundances (*p* = 0.069), and OG had significantly fewer individuals compared to CN (*p* = 0.002) (Figure 2c, Table S1). *Poecilus cupreus* was significantly more abundant in CN, CF and NG compared to OG (*p* < 0.001 for all comparisons with OG). NG had significantly lower individual numbers than CF (*p* = 0.001), while CN and CF did not differ significantly (*p* = 0.469) (Figure 2d, Table S1). The highest abundance of *Pterostichus melanarius* was found in CF, which differed significantly from all other habitat types (*p* < 0.01), indicating a strong positive effect of CF on this species. Additionally, OG had significantly fewer individuals than both CN and NG (OG vs. CN: *p* = 0.037; OG vs. NG: *p* = 0.004), while abundance did not differ between CN and NG (*p* = 0.784) (Figure 2e, Table S1).

No distance effects were observed for any carabid species between adjacent habitat types (OG-NG, OG-CN, OG-CF).

**Figure 3:**
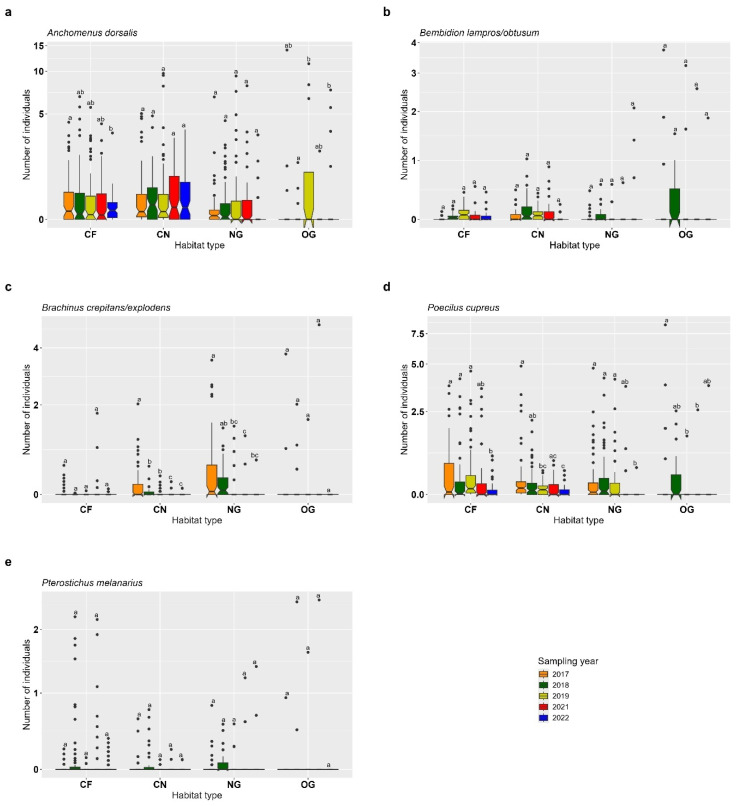
Year-effect across old semi-natural grassland (OG), newly established grassland strips (NG), cereal fields near (CN) and cereal fields far (CF) on common epigean carabids. (**a**) *Anchomenus dorsalis*, (**b**) *Bembidion lampros*/*obtusum*, (**c**) *Brachinus crepitans*/*explodens*, (**d**) *Poecilus cupreus*, (**e**) *Pterostichus melanarius*. Box-plots show the median, notches, 25% and 75% percentiles and outliers (●). Indicator letters reveal significant differences between habitat types. Habitat types sharing the same letters are not significantly different (Tukey HSD, significance level *p* < 0.05). The figure is log-transformed on the y-axes. Number of individuals are total count data per sampling plot.

For *Anchomenus dorsalis*, no significant differences in abundance were found across sampling years in CN and NG. In contrast, a significant decrease in abundance was observed in CF between 2017 and 2022 (*p* < 0.001), suggesting a temporal decline. In OG, individual numbers were lower in 2018 compared to both 2019 and 2022 (*p* < 0.05) (Figure 3a, Table S2). The abundance of *Bembidion lampros/obtusum* remained stable across all sampling years in each of the four habitat types, with no significant differences detected (all *p* > 0.05) (Figure 3b, Table S2). For *Brachinus crepitans*/*explodens*, no significant temporal differences in abundance were found in OG and CF. However, in CN and NG, higher individual numbers were recorded in 2017 compared to 2019–2022 (CN 2017 vs. 2019–2022: *p* < 0.05; NG 2017 vs. 2019–2022: *p* < 0.05), suggesting a decline in abundance in these habitats over time (Figure 3c, Table S2). *Poecilus cupreus* showed a clear temporal decline in CF, CN and NG, with significantly lower individual numbers recorded in 2022 compared to 2017 (CF: *p* = 0.014; CN: *p* < 0.001; NG: *p* < 0.001) (Figure 3d, Table S2). The abundance of *Pterostichus melanarius* remained unchanged across all sampling years and habitats, with no significant differences detected (all *p* > 0.05) (Figure 3e, Table S2).

### 3.2. Spiders

**Figure 4:**
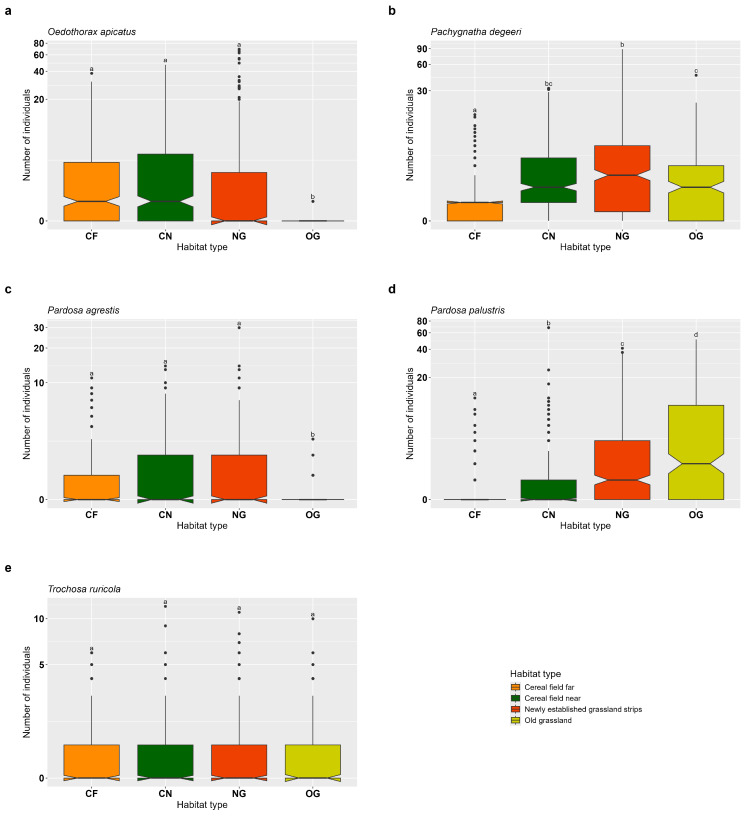
Effects of habitat type on common epigean spiders in old semi-natural grassland (OG), newly established grassland strips (NG), cereal fields near (CN) and cereal fields far (CF). (**a**) *Oedothorax apicatus*, (**b**) *Pachygnatha degeeri*, (**c**) *Pardosa agrestis*, (**d**) *Pardosa palustris*, (**e**) *Trochosa ruricola*. Box-plots show the median, notches, 25% and 75% percentiles and outliers (●). Indicator letters reveal significant differences between habitat types. Habitat types sharing the same letters are not significantly different (Tukey HSD, significance level *p* < 0.05). The figure is log-transformed on the y-axes. Number of individuals are total count data per sampling plot.

The individual numbers of *Oedothorax apicatus* and *Pardosa agrestis* were significantly higher in CF, CN and NG compared to OG, where the lowest abundances were recorded (*O. apicatus* and *P. agrestis*: CF vs. OG: *p* < 0.001; CN vs. OG: *p* < 0.001; NG vs. OG: *p* < 0.001). This indicates a negative effect of OG on the abundance of these two species, while CF, CN and NG show positive effects in comparison. No significant differences were found among CF, CN and NG (all *p* > 0.05) (Figure 4a,c, Table S3). For *Pachygnatha degeeri*, non-significant differences were observed between CN and NG (*p* = 0.566) and between OG and CN (*p* = 0.258). However, a significantly lower abundance was recorded in CF compared to OG, NG and CN (CF vs. OG: *p* < 0.001; CF vs. CN: *p* < 0.001; CF vs. NG: *p* < 0.001), suggesting a negative effect of CF on this species (Figure 4b, Table S3). The abundance of *Pardosa palustris* differed significantly across all habitat types (all *p* < 0.001), with the highest number of individuals found in OG (Figure 4d, Table S3). For *Trochosa ruricola*, no significant differences in abundance were found among the four habitat types (all *p* > 0.05) (Figure 4e, Table S3).

**Figure 5:**
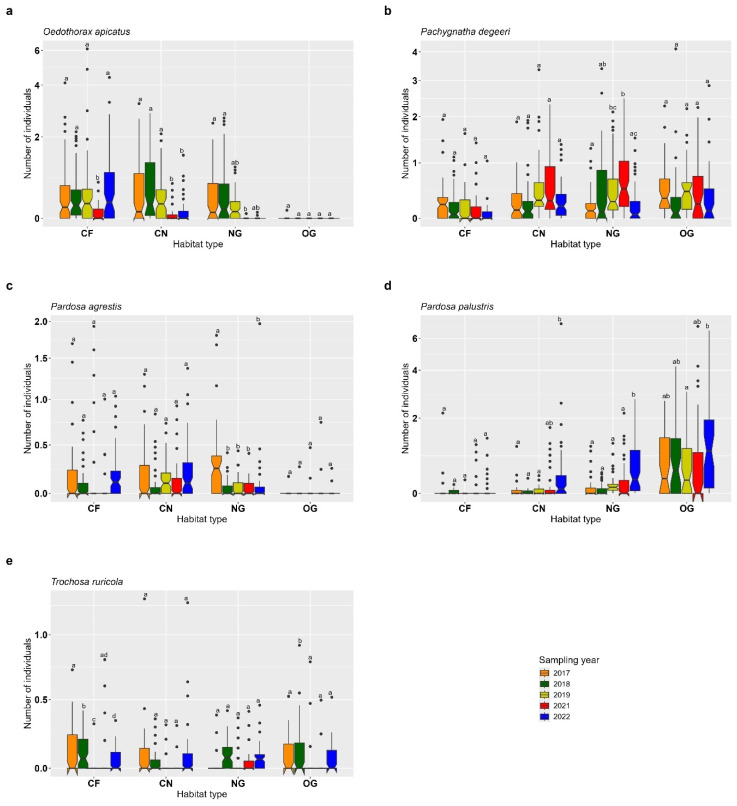
Year-effect across old semi-natural grassland (OG), newly established grassland strips (NG), cereal fields near (CN) and cereal fields far (CF) on common epigean spiders. (**a**) *Oedothorax apicatus*, (**b**) *Pachygnatha degeeri*, (**c**) *Pardosa agrestis*, (**d**) *Pardosa palustris*, (**e**) *Trochosa ruricola*.Box-plots show the median, notches, 25% and 75% percentiles and outliers (●). Indicator letters reveal significant differences between habitat types. Habitat types sharing the same letters are not significantly different (Tukey HSD, significance level *p* < 0.05). The figure is log-transformed on the y-axes. Number of individuals are total count data per sampling plot.

For *Oedothorax apicatus*, abundance showed a decreasing trend in 2021 compared to previous years, particularly in CF, where the lowest number of individuals was observed (2017 vs. 2021: *p* = 0.0003). Similarly, in CN, abundance was significantly lower in 2021 and 2022 compared to earlier years (2017 vs. 2021: *p* < 0.0001; 2017 vs. 2022: *p* = 0.0006). In NG, abundance significantly differed between 2017 and 2021 (*p* = 0.032), with a drop in 2021. In OG, no significant differences in abundance were found among sampling years (all *p* > 0.05) (Figure 5a, Table S4). For *Pachygnatha degeeri*, significant differences were detected in NG, with lower abundance in 2019–2021 compared to 2017 (2017 vs. 2019: *p* = 0.025; 2017 vs. 2021: *p* < 0.0001) and another significant change between 2021 and 2022 (*p* = 0.001). In CF, CN and OG, no significant temporal differences were found (all *p* > 0.05), indicating relative temporal stability (Figure 5b, Table S4). For *Pardosa agrestis*, no significant differences in abundance were observed across the six sampling years in CF, CN and OG (all *p* > 0.05). In NG, however, the highest number of individuals was recorded in 2017, followed by a notable decline in the subsequent years (2017 vs. 2018: *p* = 0.001; 2017 vs. 2019: *p* = 0.001; 2017 vs. 2021: *p* = 0.0009; 2017 vs. 2022: *p* = 0.002) (Figure 5c, Table S4). *Pardosa palustris* showed significant temporal variation in CN (2017 vs. 2022: *p* = 0.005; 2018 vs. 2022: *p* = 0.003; 2019 vs. 2022: *p* = 0.005), NG (2017 vs. 2022: *p* < 0.0001; 2018 vs. 2022: *p* < 0.0001; 2019 vs. 2022: *p* = 0.0007; 2021 vs. 2022: *p* = 0.001) and OG (2019 vs. 2022: *p* = 0.034) (Figure 5d, Table S4). For *Trochosa ruricola*, significant differences in abundance were found between sampling years in CF (2017 vs. 2018: *p* < 0.0001; 2017 vs. 2019: *p* = 0.025; 2017 vs. 2022: *p* = 0.016; 2018 vs. 2019, 2021, 2022: all *p* < 0.0001; 2019 vs. 2021: *p* = 0.005; 2019 vs. 2022: *p* < 0.0001) and OG (2018 vs. 2017, 2019, 2021, 2022: all *p* < 0.0001), indicating temporal variation. In CN and NG, no significant differences were observed (all *p* > 0.05), suggesting relatively consistent abundance levels (Figure 5e, Table S4).

**Figure 6:**
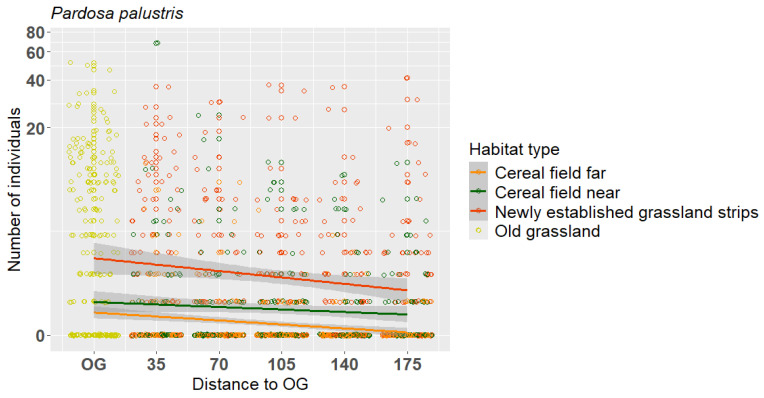
Distance effect on the abundance of the spider *Pardosa palustris* between old semi-natural grassland (OG, yellow circles) and the adjacent habitat types, newly established grassland (NG, red circles), cereal near (CN, green circles) and cereal far (CF, orange circles). The figure is log-transformed on the y-axes. Numberof individuals are total count data per sampling plot.

A significant distance effect from OGs into NG, CF and CN was observed for *P. palustris* with a significant decrease in number of individuals towards the field interior (*p* < 0.0001; Figure 6). No distance effects were observed for any other spider species.

## 4. Discussion

Most of the studied carabid species were strongly associated with cereal fields, which also confirms the results on the general abundance of carabids, measured at family level, in the study region [14]. Of all epigean carabids examined, *Anchomenus dorsalis* and *Poecilus cupreus* were the most numerous epigean carabids in the present study. While they were only found in small numbers in old semi-natural grassland, their main distribution was within cereal fields and grassland strips. As eurytopic species, *A. dorsalis* and *P. cupreus* occur in a large variety of habitat types, including semi-natural habitats such as field margins [30], but they are also very common on arable land in European agroecosystems [31,32,33]. However, grassland strips may serve as an additional food source and overwintering site for both species [30].

*Bembidion lampros/obtusum* clearly preferred cereal fields over both grassland habitats. This result is in line with Hoffmann et al. [34] who observed that the number of individuals of *Bembidion* species decreases as soon as grassland strips replace arable land. The higher ground vegetation cover in old semi-natural grassland and newly established grassland strips compared to cereal fields probably represents a movement barrier for these small epigean carabids and is therefore less attractive for *Bembidion* species. This is also consistent with the results of Ranja and Irmler [35], who observed a lower number of small-sized carabid species in field margins and grassland strips than in arable fields.

*Pterostichus melanarius* was found almost exclusively in cereal fields and newly established grassland strips, with the highest activity-densities in cereal fields far away from the newly established grassland strips. Similar to the other epigean carabids studied, the lowest activity was found in old semi-natural grassland. *P. melanarius* utilizes various habitats [36], is often found as a dominant species in arable crops and can cope with a high disturbance rate in cereal fields [37]. Considering the seasonal activity of *P. melanarius* as an autumn breeder [38] may explain its overall lower number of individuals in our studied habitats since we conducted sampling in spring.

Highest number of individuals of *Brachinus crepitans*/*explodens* was found in grassland strips, and higher number of individuals were observed in 2017 than in 2019–2022. Habitat conditions in the initial years after establishment of the grassland strips may have promoted these species, but habitat suitability might have degraded when grassland strips were getting older. A declining amount of open ground [39] and increasing vegetation cover [35] might have been the reason for the decline of *Brachinus crepitans*/*explodens* over the sampling years. Furthermore, similar activity-densities in NG and CN suggest an interaction between grassland strips and adjacent cereals.

*Oedothorax apicatus* and *Pardosa agrestis*, typical epigean spiders within various crops [40], were almost exclusively found in cereal fields and adjacent grassland strips. Both species use semi-natural habitats for overwintering [41], from where they can migrate into the adjacent cereal fields in spring [42]. Consequently, grassland strips along cereal fields may support an effective colonization of adjacent crop fields. Further, for *O. apicatus*, which disperses predominantly over the soil surface, the proximity to an adjacent semi-natural habitat is necessary for an effective colonization of adjacent arable crops [42,43]. Especially due to their dispersal mode, cursorial spiders can reach high activity densities in crops adjacent to semi-natural habitats [44] which likely makes this habitat an important source for beneficial spiders. Some of the epigean spiders investigated were primarily found in old semi-natural grassland. This was especially true for *Pardosa palustris*, *Trochosa ruricola* and *Pachygnatha degeeri*. *P. palustris* and *P. degeeri* are typical inhabitants of grassland habitats but are also able to colonize crop fields in larger numbers [45]. Öberg et al. [46] showed that *P. palustris* revealed a clear preference for field margins and thus preferring grassland over arable land. This is in line with the present findings with a high dominance of *P. palustris* in both grassland habitats. In addition, *P. palustris* showed a distribution pattern with the highest individual numbers in OG and decreasing numbers toward the adjacent habitats CN, CF and NG. In 2022, higher abundances of *P. palustris* were recorded in cereal fields compared to 2017. While this might suggest a broader distribution or changes in habitat association over time, our data do not allow for conclusions about individual movement or density, as our pitfalls only reflect local abundance and not actual dispersal. Therefore, the observed pattern could also be influenced by other factors such as interannual variation in weather and local habitat conditions. We acknowledge that long-term differences in annual species like *P. palustris* are difficult to interpret in terms of direct ecological processes, but the distribution pattern of *P. palustris* can cautiously be interpreted as spillover between two semi-natural habitats and also between semi-natural habitats and cereals.

Further, Schmidt and Tscharntke [42] suggested an affinity of *P. degeeri* for perennial habitats which is in line with our results. Individual numbers of *P. degeeri* were higher in both grassland habitats and cereals next to the grassland strip. Higher numbers in the grassland strips suggest a spill-over of individuals between grassland strips and adjacent cereals. The number of *Trochosa ruricola* individuals were evenly distributed across the habitat types studied. Although Schmidt et al. [47] revealed that this species is positively affected by higher amounts of non-crop habitats in the immediate surroundings, no clear distribution pattern could be found in the present investigation.

## 5. Conclusions

In conclusion, the study highlights the strong association of most carabid species with cereal fields, with species such as *Anchomenus dorsalis* and *Poecilus cupreus* being particularly abundant in these habitats. While some species, like *Bembidion lampros/obtusum*, favored cereal fields over grassland habitats, others, such as *Brachinus crepitans*/*explodens*, showed a preference for younger grassland strips, with their numbers declining as these habitats aged. Additionally, epigean spiders like *Oedothorax apicatus* and *Pardosa agrestis* were primarily found in cereal fields and adjacent grassland strips, underscoring the importance of semi-natural habitats for supporting beneficial species in agricultural landscapes.

## Data Availability

The original contributions presented in this study are included in the article/Supplementary Material. Further inquiries can be directed to the corresponding author.

## Supplementary Materials

The following supporting information can be downloaded at: https://www.mdpi.com/article/10.3390/insects16050439/s1, Table S1: Results obtained from generalized linear mixed models (GLMM) showing the effects of habitat type on common carabids in old semi-natural grassland (OG), newly established grassland strips (NG), cereals near (CN) and cereal far (CF). Significant *p*-values are highlighted in bold; Table S2: Results obtained from generalized linear mixed models (GLMM) showing year-wise comparisons of the abundance of common arable carabids in old semi-natural grassland (OG), newly established grassland strips (NG), cereals near (CN) and cereal far (CF) among sampling years. Significant *p*-values are highlighted in bold; Table S3: Results obtained from generalized linear mixed models (GLMM) showing the effects of habitat type on common spiders in old semi-natural grassland (OG), newly established grassland strips (NG), cereals near (CN) and cereal far (CF). Significant *p*-values are highlighted in bold; Table S4: Results obtained from generalized linear mixed models (GLMM) showing year-wise comparisons of the abundance of common arable spiders in old semi-natural grassland (OG), newly established grassland strips (NG), cereals near (CN) and cereal far (CF) among sampling years. Significant *p*-values are highlighted in bold.
