# Supplementary material for: The Potential of Newly Established Grassland Strips and Permanent Semi-Natural Grassland to Promote Common Carabids and Spiders on Arable Land
Source: Insects. 2025 Apr 22;16(5):439. doi: 10.3390/insects16050439 (PMC12112026; doi:10.3390/insects16050439)
Supplement: Supplementary file 1 [file insects-16-00439-s001.zip › insects-3512967-supplementary.pdf]

Supplementary material

Table S1. Results obtained from Generalized Linear Mixed Models (GLMM) showing the effects of habitat type on common carabids in old semi-natural grassland (OG), newly established grassland strips (NG), cereals near (CN) and cereal far (CF). Significant p-values are highlighted in bold.

**Carabids**					
Anchomenus dorsalis	Habitat type	Estimates	Std. error	z-value	p-value
	CF (Intercept)	1.5982	0.5863	2.726	**0.006**
	CN	0.1142	0.2333	0.489	0.625
	NG	-1.3890	0.2604	-5.335	**<0.0001**
	OG	-3.3712	0.2372	-14.211	**<0.0001**
Bembidion lampros/obtusum	Habitat type	Estimates	Std. error	z-value	p-value
	CF (Intercept)	-0.1102	0.3704	-0.297	**0.766**
	CN	0.0114	0.3488	0.033	0.974
	NG	-1.2259	0.3630	-3.377	**0.0007**
	OG	-1.3588	0.3236	-4.199	**<0.0001**
Brachinus crepitans/explodens	Habitat type	Estimates	Std. error	z-value	p-value
	CF (Intercept)	-2.777	0.6052	-4.589	**<0.0001**
	CN	0.9111	0.5444	1.674	0.094
	NG	2.1155	0.5429	3.897	**<0.0001**
	OG	-0.7920	0.4914	-1.612	0.107
Poecilus cupreus	Habitat type	Estimates	Std. error	z-value	p-value
	CF (Intercept)	1.4486	04913	2.949	**0.003**
	CN	-0.7100	0.4751	-1.494	0.135
	NG	-1.3097	0.4759	-2.752	**0.006**
	OG	-3.0390	0.3837	-7.920	**<0.0001**
Pterostichus melanarius	Habitat type	Estimates	Std. error	z-value	p-value
	CF (Intercept)	-0.5226	0.9604	-0.544	0.586
	CN	-2.8142	0.8014	-3.512	**0.0004**
	NG	-2.6402	0.9005	-2.932	**0.003**
	OG	-4.1682	0.7236	-5.760	**<0.0001**

Table S2. Results obtained from Generalized Linear Mixed Models (GLMM) showing year-wise comparisons of the abundance of common arable carabids in old semi-natural grassland (OG), newly established grassland strips (NG), cereals near (CN) and cereal far (CF) among sampling years. Significant p-values are highlighted in bold.

**Carabids-Year**					
Anchomenus dorsalis	OG	Estimates	Std. error	z-value	p-value
2017 (Intercept)		0.335	0.733	0.457	0.647
2018		-1.543	0.667	-2.131	**0.021**
2019		0.405	0.547	0.741	0.459
2021		-0.317	0.571	-0.556	0.577
2022		0.263	0.548	0.480	0.631
	CN	Estimates	Std. error	z-value	p-value
2017 (Intercept)		-0.244	0.162	-1.510	0.131

2018		-0.095	0.232	-0.408	0.683
2019		0.254	0.220	1.152	0.249
2021		-0.006	0.229	-0.027	0.978
2022		-0.082	0.232	-0.355	0.722
	CF	Estimates	Std. error	z-value	p-value
2017 (Intercept)		-0.370	0.160	-2.315	**0.020**
2018		0.021	0.225	0.095	0.924
2019		0.060	0.228	-0.265	0.791
2021		-0.158	0.233	-0.678	0.498
2022		-0.616	0.260	-2.370	**0.017**
	NG	Estimates	Std. error	z-value	p-value
2017 (Intercept)		-1.928	0.305	-6.314	**<0.0001**
2018		0.488	0.374	1.303	0.192
2019		0.630	0.369	1.706	0.088
2021		0.348	0.380	0.915	0.360
2022		-0.142	0.405	-0.352	0.725
Brachinus crepitans/explodens	OG	Estimates	Std. error	z-value	p-value
2017 (Intercept)		-5.980	1.522	-3.928	**<0.0001**
2018		0.174	1.331	0.131	0.896
2019		-0.629	1.561	-0.403	0.687
2021		-1.102	1.800	-0.612	0.540
2022		-19.776	17202.40	-0.001	0.999
	CN	Estimates	Std. error	z-value	p-value
2017 (Intercept)		1.686	0.077	21.636	**<0.0001**
2018		-1.159	0.186	-6.214	**<0.0001**
2019		-1.288	0.244	-5.275	**<0.0001**
2021		-2.688	0.372	-7.214	**<0.0001**
2022		-3.624	0.537	-6.746	**<0.0001**
	CF	Estimates	Std. error	z-value	p-value
2017 (Intercept)		-3.471	0.655	-5.299	**<0.0001**
2018		-2.345	2.215	-1.059	0.290
2019		-1.246	1.386	-0.899	0.368
2021		0.420	0.843	0.498	0.618
2022		-1.644	1.628	-1.010	0.313
	NG	Estimates	Std. error	z-value	p-value
2017 (Intercept)		-0.831	0.175	-4.752	**<0.0001**
2018		-0.672	0.301	-2.233	**0.025**
2019		-1.356	0.386	-3.508	**0.0004**
2021		-2.594	0.663	-3.907	**<0.0001**
2022		-2.240	0.564	-3.969	**<0.0001**
Pterostichus melanarius	OG	Estimates	Std. error	z-value	p-value
2017 (Intercept)		-3.181	0.731	-4.349	**<0.0001**
2018		1.089	0.845	1.288	0.197
2019		-0.147	1.074	-0.137	0.890

2021		1.702	0.795	2.141	**0.032**
2022		-19.643	13483.58	-0.001	0.998
	CN	Estimates	Std. error	z-value	p-value
2017 (Intercept)		-3.045	0.529	-5.753	<**0.0001**
2018		0.424	0.680	0.326	0.534
2019		-2.449	1.877	-1.305	0.190
2021		-0.941	0.999	-0.942	0.346
2022		-1.574	1.278	-1.232	0.218
	CF	Estimates	Std. error	z-value	p-value
2017 (Intercept)		-3.687	0.729	-5.052	<**0.0001**
2018		1.864	0.784	2.377	**0.017**
2019		-0.455	1.171	-0.389	0.697
2021		1.583	0.801	1.975	0.053
2022		0.384	0.946	0.406	0.684
	NG	Estimates	Std. error	z-value	p-value
2017 (Intercept)		-3.156	0.559	-5.641	<**0.0001**
2018		0.471	0.713	0.661	0.509
2019		-0.096	0.811	-0.119	0.905
2021		0.541	0.703	0.769	0.442
2022		-0.139	0.820	-0.170	0.865
Bembidion lampros/obtusum	OG	Estimates	Std. error	z-value	p-value
2017 (Intercept)		-1.166	0.345	-3.373	**0.0008**
2018		-0.327	0.534	-0.613	0.540
2019		-0.082	0.499	-0.166	0.868
2021		-1.005	0.668	-1.505	0.133
2022		-2.024	1.012	-2.000	0.050
	CN	Estimates	Std. error	z-value	p-value
2017 (Intercept)		-3.030	0.556	-5.455	<**0.0001**
2018		0.955	0.578	1.651	0.098
2019		0.315	0.646	0.488	0.625
2021		0.342	0.643	0.532	0.594
2022		-0.741	0.865	-0.857	0.391
	CF	Estimates	Std. error	z-value	p-value
2017 (Intercept)		-4.110	0.901	-4.558	<**0.0001**
2018		0.958	1.056	0.933	0.351
2019		1.672	0.982	1.702	0.088
2021		1.097	1.041	1.053	0.292
2022		0.526	1.137	0.463	0.643
	NG	Estimates	Std. error	z-value	p-value
2017 (Intercept)		-3.675	0.726	-5.057	<**0.0001**
2018		0.728	0.873	0.834	0.404
2019		0.490	0.910	0.539	0.590
2021		0.446	0.918	0.486	0.627
2022		1.269	0.811	1.564	0.118

Poecilus cupreus	OG	Estimates	Std. error	z-value	p-value
2017 (Intercept)		0.825	0.246	3.348	**0.0008**
2018		-1.024	0.380	-2.690	**0.007**
2019		-2.035	0.696	-2.924	**0.003**
2021		-2.225	0.762	-2.919	**0.003**
2022		-1.750	0.715	-2.447	**0.014**
	CN	Estimates	Std. error	z-value	p-value
2017 (Intercept)		-0-926	0.183	-5.050	**<0.0001**
2018		-0.381	0.288	-1.324	0.185
2019		-0.967	0.349	-2.766	**0.005**
2021		-0.862	0.336	-2.560	**0.010**
2022		-1.796	0.486	-3.692	**0.0003**
	CF	Estimates	Std. error	z-value	p-value
2017 (Intercept)		-0.0664	0.179	-3.711	**0.0002**
2018		-0.343	0.273	-1.256	0.290
2019		-0.075	0.257	-0.291	0.770
2021		-0.508	0.285	-1.784	0.074
2022		-1.526	0.397	-3.846	**0.0001**
	NG	Estimates	Std. error	z-value	p-value
2017 (Intercept)		1.056	0.214	-4.915	**<0.0001**
2018		0.090	0.298	0.303	0.761
2019		-0.236	0.320	-0.739	0.459
2021		-0.865	0.381	-2.272	**0.023**
2022		-2.932	0.879	-3.333	**0.0008**

Table S3. Results obtained from Generalized Linear Mixed Models (GLMM) showing the effects of habitat type on common spiders in old semi-natural grassland (OG), newly established grassland strips (NG), cereals near (CN) and cereal far (CF). Significant p-values are highlighted in bold.

**Spiders**					
Pardosa agrestis	Habitat type	Estimates	Std. error	z-value	p-value
	CF (Intercept)	-0.5874	0.5677	-1.035	0.301
	CN	0.3593	0.4025	0.893	0.372
	NG	0.4852	0.4081	1.189	0.234
	OG	-2.3714	0.4169	-5.688	**<0.0001**
Pardosa palustris	Habitat type	Estimates	Std. error	z-value	p-value
	CF (Intercept)	-1.8829	0.5332	-3.531	**0.0004**
	CN	0.6256	0.2309	2.709	**0.007**
	NG	1.9906	0.2145	9.281	**<0.0001**
	OG	3.0896	0.2109	14.653	**<0.0001**
Oedothorax apicatus	Habitat type	Estimates	Std. error	z-value	p-value
	CF (Intercept)	0.9016	0.7624	1.182	0.237
	CN	0.3599	0.9328	0.386	0.700
	NG	-0.9996	0.9169	-1.090	0.276

	OG	-6.3512	1.0201	-6.226	**<0.0001**
Pachygnatha degeeri	Habitat type	Estimates	Std. error	z-value	p-value
	CF (Intercept)	0.0465	0.3217	0.144	0.885
	CN	0.9665	0.1790	5.400	**<0.0001**
	NG	1.1422	0.1948	5.863	**<0.0001**
	OG	0.8052	0.1662	4.846	**<0.0001**
Trochosa ruricola	Habitat type	Estimates	Std. error	z-value	p-value
	CF (Intercept)	-1.2224	0.6515	-1.876	0.060
	CN	-0.2699	0.3553	-0.760	0.447
	NG	0.0371	0.3442	0.108	0.914
	OG	-0.0439	0.2556	-0.172	0.863

Table S4. Results obtained from Generalized Linear Mixed Models (GLMM) showing year-wise comparisons of the abundance of common arable spiders in old semi-natural grassland (OG), newly established grassland strips (NG), cereals near (CN) and cereal far (CF) among sampling years. Significant p-values are highlighted in bold.

**Spiders-Year**					
Oedothorax apicatus	OG	Estimates	Std. error	z-value	p-value
2017 (Intercept)		<0.0001	<0.0001	-2.872	**0.004**
2018		<0.0001	<0.0001	0	0.999
2019		<0.0001	<0.0001	0	0.999
2021		<0.0001	<0.0001	0	0.999
2022		<0.0001	<0.0001	0	0.999
	CN	Estimates	Std. error	z-value	p-value
2017 (Intercept)		-0.566	0.153	-3.695	**0.0002**
2018		0.189	0.207	0.916	0.359
2019		-0.316	0.236	-1.340	0.180
2021		-2.184	0.481	-4.533	**<0.0001**
2022		-1.327	0.334	-3.965	**<0.0001**
	CF	Estimates	Std. error	z-value	p-value
2017 (Intercept)		-0.701	0.167	-4.191	**<0.0001**
2018		-0.017	0.237	-0.075	0.940
2019		0.139	0.229	0.609	0.542
2021		-1.251	0.350	-3,567	**0.0003**
2022		0.319	0.220	1.450	0.147
	NG	Estimates	Std. error	z-value	p-value
2017 (Intercept)		-0.861	0.177	-4.849	**<0.0001**
2018		0.114	0.244	0.470	0.638
2019		-0.639	0.302	-2.115	**0.034**
2021		-4.341	1.566	-2.771	**0.005**
2022		-4.794	1.960	-2.445	**0.014**
Pardosa agrestis	OG	Estimates	Std. error	z-value	p-value
2017 (Intercept)		-4.164	1.195	-3.482	**0.0004**
2018		-0.934	2.251	-0.415	0.678
2019		0.106	1.737	-0.061	0.951
2021		0.755	1.449	0.521	0.602

2022		0.104	1.648	0.063	0.949
	CN	Estimates	Std. error	z-value	p-value
2017 (Intercept)		-1.638	0.261	-6.254	**<0.0001**
2018		-0.954	0.496	-1.921	0.054
2019		-0.319	0.403	-0.792	0.428
2021		-0.557	0.434	-1.284	0.199
2022		0.095	0.361	0.264	0.792
	CF	Estimates	Std. error	z-value	p-value
2017 (Intercept)		-1.786	0.282	-6.333	**<0.0001**
2018		-0.693	0.488	-1.149	0.155
2019		-0.109	0.410	-0.268	0.789
2021		-1.044	0.551	-1.889	0.058
2022		-0.008	0.399	-0.021	0.983
	NG	Estimates	Std. error	z-value	p-value
2017 (Intercept)		-1.173	0.207	-5.562	**<0.0001**
2018		-1.858	0.565	-3.287	**0.001**
2019		-1.526	0.491	-3.106	**0.001**
2021		-1.888	0.572	-3.297	**0.0009**
2022		-1.368	0.461	-2.969	**0.003**
Trochosa ruricola	OG	Estimates	Std. error	z-value	p-value
2017 (Intercept)		-0.628	0.204	-3.080	**0.002**
2018		1.041	0.237	4.386	**<0.0001**
2019		-0.233	0.307	-0.761	0.446
2021		-0.875	0.376	-2.326	**0.020**
2022		-0.233	0.307	-0.761	0.446
	CN	Estimates	Std. error	z-value	p-value
2017 (Intercept)		-2.756	0.458	-6.016	**<0.0001**
2018		-0.074	0.660	-0.114	0.910
2019		-0.820	0.828	-0.990	0.322
2021		-0.249	0.692	-0.361	0.718
2022		0.596	0.570	1.046	0.296
	CF	Estimates	Std. error	z-value	p-value
2017 (Intercept)		-1.139	0.223	-5.097	**<0.0001**
2018		1.697	0.256	6.610	**<0.0001**
2019		-1.386	0.474	-2.923	**0.003**
2021		0.189	0.304	0.621	0.534
2022		0.847	0.276	3.070	**0.002**
	NG	Estimates	Std. error	z-value	p-value
2017 (Intercept)		-3.147	0.557	-5.560	**<0.0001**
2018		0.703	0.681	1.033	0.302
2019		-0.385	0.875	-0.440	0.660
2021		-0.007	0.789	-0.009	0.993
2022		0.582	0.695	0.838	0.402
Pachygnatha degeeri	OG	Estimates	Std. error	z-value	p-value

2017 (Intercept)		-0.698	0.211	-3.305	**0.0009**
2018		-0.386	0.332	-1.163	0.244
2019		0.059	0.294	0.201	0.840
2021		0.030	0.296	0.104	0.917
2022		-0.425	0.336	-1.266	0.205
	CN	Estimates	Std. error	z-value	p-value
2017 (Intercept)		-1.311	0.222	-5.896	**<0.0001**
2018		-0.037	0.317	-0.117	0.906
2019		0.638	0.275	2.322	**0.020**
2021		0.706	0.271	2.600	**0.009**
2022		0.033	0.312	0.107	0.915
	CF	Estimates	Std. error	z-value	p-value
2017 (Intercept)		-1.331	0.224	-5.926	**<0.0001**
2018		-0.255	0.340	-0.751	0.452
2019		-0.483	0.363	-1.328	0.184
2021		-0.417	0.356	-1.170	0.242
2022		-1.068	0.444	-2.404	**0.016**
	NG	Estimates	Std. error	z-value	p-value
2017 (Intercept)		-1.595	0.256	-6.223	**<0.0001**
2018		0.761	0.310	2.450	**0.014**
2019		0.898	0.304	2.952	**0.003**
2021		1.239	0.291	4.257	**<0.0001**
2022		0.264	0.341	0.775	0.438
Pardosa palustris	OG	Estimates	Std. error	z-value	p-value
2017 (Intercept)		-0.341	0.202	-1.687	0.091
2018		0.172	0.277	0.621	0.534
2019		-0.106	0.291	-0.363	0.716
2021		0.067	0.282	0.241	0.809
2022		0.650	0.258	2.517	**0.011**
	CN	Estimates	Std. error	z-value	p-value
2017 (Intercept)		-3.061	0.493	-6.205	**<0.0001**
2018		-0.731	0.839	-0.871	0.383
2019		0.010	0.679	0.015	0.988
2021		1.241	0.551	2.248	**0.024**
2022		1.799	0.527	3.412	0.0006
	CF	Estimates	Std. error	z-value	p-value
2017 (Intercept)		-2.793	0.466	-5.985	**<0.0001**
2018		-0.575	0.778	-0.740	0.459
2019		-1.272	0.997	-1.275	0.202
2021		0.608	0.580	1.049	0.294
2022		0.187	0.631	0.297	0.766
	NG	Estimates	Std. error	z-value	p-value
2017 (Intercept)		-2.022	0.317	-6.371	**<0.0001**
2018		-0.546	0.524	-1.042	0.298

2019	0.044	0.407	1.082	0.279
2021	0.483	0.403	1.199	0.231
2022	1.584	0.348	4.574	**<0.0001**
